# Idiopathic Tetanus in a Horse, Successfully Treated by Chloroform

**Published:** 1849-03

**Authors:** 


*Idiopathic Tetanus in a Horse, successfully treated by Chloroform.* By T. L. Maddin, Student of Medicine in the University of Louisville.

In August last I tried the effects of chloroform upon a horse affected with Lock-jaw, and also violent convulsions of the entire muscular system. The circumstances of the case were these:—A farmer, residing in North Alabama, at whose house I was living, discovered, early in the morning, that one of his horses was very singularly affected, and, upon examination, found that he had tetanus, or lock-jaw; and after a short time tetanic convulsions of the entire body supervened—which continued to grow more violent during the day.

Having been absent from home all day, when I arrived at his house late in the evening, my landlord remarked that he had a horse dying, and also mentioned his symptoms.

The opportunity, I conceived, was an excellent one to test the efficacy of chloroform, and immediately I suggested the trial of the remedy. The owner of the horse remarked that he did not appear as if he could live longer than ten minutes, but that I was at liberty to do as I pleased.

The horse was down, and could not raise his head; his limbs were in an extreme state of rigidity, and his jaws firmly clenched. I first gave him half an ounce of laudanum, with twenty-five grains of camphor dissolved in it. This did not make any impression upon the symptoms whatever. I then caused him to breathe chloroform, and in less than two minutes he was fully under the influence of it. He remained thus for fifteen minutes; during which time his limbs were quite flexible; his muscular system generally relaxed; and his jaws could be opened about two inches. At the expiration of this time, there were symptoms of a return of the convulsions. I brought him rapidly under the influence of the chloroform again, and thus warded them off. It was now twenty minutes before the anæsthesia passed off, and it was then found that he was able to get up and walk about. In less than three hours from this time he was grazing about the lot, and next morning appeared perfectly well.

I have believed that an account of this case would be acceptable to the profession, notwithstanding that the subject of it was a horse, for it shows very conclusively the perfect control which this potent agent possesses over tetanus, and convulsive diseases generally, which are among the most intractable that the medical man has to combat.—*Western Journal of Medicine and Surgery.*

